# CT-based vascular invasion in pancreatic ductal adenocarcinoma compared with intraoperative and histological findings

**DOI:** 10.1186/s13244-026-02318-0

**Published:** 2026-06-21

**Authors:** Gala Nacul Mora, Andreas Andreou, Ziad Maksoud, Felix Becker, Barbara Heitplatz, Eva Wardelmann, Benjamin Strücker, Shadi Katou, Haluk Morgül, Andreas Pascher, Michael Köhler, Gesa Helen Pöhler

**Affiliations:** 1 Department of Radiology, University Hospital Münster, Münster, Germany; 2 Department of General, Visceral and Transplant Surgery, University Hospital Münster, Münster, Germany; 3 Department of Pathology, University Hospital Münster, Münster, Germany

**Keywords:** Pancreatic neoplasms, Tomography (X-ray computed), Neoplasm invasiveness, Sensitivity and specificity

## Abstract

**Purpose:**

To assess the concordance of CT-based radiological, surgical, and histopathological determination of vascular invasion in pancreatic ductal adenocarcinoma (PDAC).

**Materials and methods:**

This retrospective single-center study included 103 treatment-naive PDAC patients (median age 68 years, male 61%) with arterial-/portal venous-contrast enhanced CT undergoing successful primary resection. Three radiologists independently assessed tumor-vessel contact according to NCCN criteria. Radiological vascular invasion was operationally defined as > 180° or contour irregularity versus no invasion defined as no contact or contact ≤ 180° without contour irregularity, to compare to binary intraoperative and histological reference. Intraoperatively, venous and arterial invasion were defined as tumor adherence to or invasion of the vessel wall. Histopathologically, venous invasion (V0/1) was defined according to UICC-TNM. Additionally, as major arteries were not resected, microscopically seen periarterial perineural invasion (Pn_art_) served as a surrogate marker for arterial invasion (Pn0/Pn1), intermodal concordance of CT-to-surgery ≤/ > 4 weeks) was compared, Cohen’s kappa and McNemar’s-tests were used.

**Results:**

Median CT-to-surgery was 17 days (81% within 4 weeks). CT-based interobserver agreement was κ = 0.8 for venous, κ = 0.6 for arterial invasion determination. Intraoperatively, 35% patients showed venous, 5% arterial involvement. Histopathologically, 14% showed venous invasion, 3% Pn_art_1-status. CT-surgery venous invasion concordance was 75%, arterial 96%. CT-histopathology venous invasion concordance was 81%, 96% for Pn_art_-status. CT-to-surgery interval ≤4 weeks showed higher CT-surgery (77% vs 65%) and CT-histopathology venous invasion concordance (84% vs 60%) compared to > 4 weeks interval.

**Conclusion:**

NCCN-derived, threshold-based radiological venous invasion determination was similar to binary intraoperative and histopathological findings and decreased with longer CT-to-surgery intervals.

**Critical relevance statement:**

NCCN-derived, threshold-based CT venous-invasion determination in PDAC is concordant with binary intraoperative and histopathological invasion, but depends on the selected threshold and decreases with longer CT-to-surgery interval, underlining the clinical need for standardized staging intervals in patients undergoing surgery.

**Key Points:**

- Comparison between CT vascular assessment and surgical/histopathological findings in PDAC remains highly complex, and current guidelines lack CT-to-surgery interval recommendations.
- Threshold-based CT venous invasion agreed moderately with surgery and strongly with histopathology, while CT-based arterial status correlated strongly with surgery, albeit in markedly low overall incidence.
- CT-vascular assessment precision decreases with longer time intervals, showing the need for establishing timeframes for preoperative imaging.

**Graphical Abstract:**

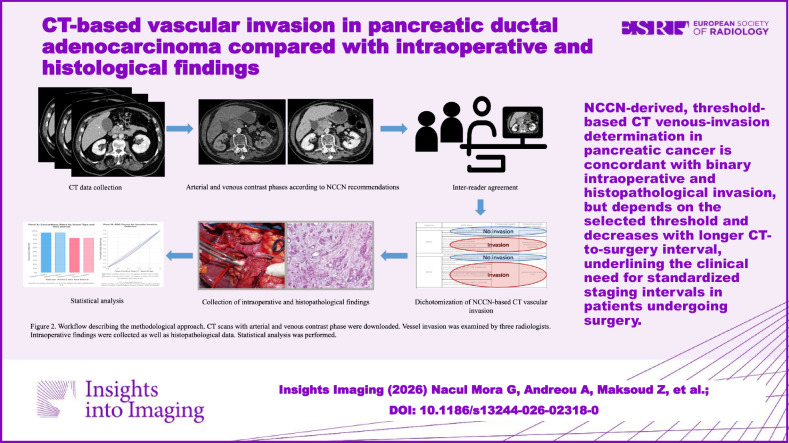

## Introduction

Pancreatic ductal adenocarcinoma (PDAC) remains one of the most lethal malignancies worldwide, with a 5-year survival rate of approximately 10–13% and an increasing incidence in Western populations [1]. The dismal prognosis is primarily attributed to late diagnosis, early metastasis, and limited surgical resectability [2]. Surgical resection is a critical component of curative-intent treatment for patients with potentially or borderline resectable PDAC [3]. However, the challenge in patient selection for surgical planning is the accurate preoperative assessment of tumor extent and vascular involvement.

The relationship between tumor and major peripancreatic vessels, including the superior mesenteric artery (SMA), superior mesenteric vein (SMV), portal vein (PV), celiac axis (CA), and common hepatic artery (CHA), determines resectability classification according to established criteria from the National Comprehensive Cancer Network (NCCN) and international consensus guidelines [4]. Precise identification of vascular invasion is essential for determining the further therapeutic strategy [5, 6].

Contrast-enhanced computed tomography (CT) with dedicated pancreatic protocol imaging is the primary imaging modality for the preoperative staging of PDAC [7]. This technique employs thin-section multidetector CT acquisition with intravenous contrast administration in arterial and portal venous phases, optimizing the visualization of all vessels [8]. CT enables the assessment of tumor size, extension, vascular involvement, lymph node status, and metastasis. Despite continuous advances in CT technology, accurate prediction of vascular invasion remains challenging. The subtle distinction between tumor abutment and true vascular infiltration, particularly in borderline resectable cases, represents a recurring diagnostic dilemma with significant therapeutic implications [9].

Previous studies investigating the concordance between radiological assessment and surgical findings have yielded variable results, with reported accuracies ranging from 60% to 90%, depending on the vessel involved and the criteria applied for defining vascular involvement [10, 11]. The sensitivity of CT for detecting vascular invasion ranges from 70% to 96%, with specificity values between 82% and 100% [12, 13]. Discrepancies between preoperative CT interpretation and intraoperative reality can lead to unnecessary exploration in unresectable cases or missed opportunities for potentially curative resections. Furthermore, the reproducibility of radiological assessment and potential interobserver variability add additional complexity to clinical decision-making, with studies demonstrating only fair to moderate agreement among experienced radiologists in applying NCCN-criteria [14].

To date, only a limited number of studies have systematically evaluated the concordance between NCCN-based CT criteria and histopathological findings [15, 16]. A critical distinction must be drawn between venous and arterial involvement, as these entities differ substantially in their imaging assessment and pathological definitions. While venous invasion can be evaluated against relatively consistent reference standards, arterial involvement remains considerably more complex and is addressed in only a few studies. Reference standards are inconsistently defined across the literature, most studies rely exclusively on intraoperative findings, while others additionally incorporate histopathology without specifying the exact criteria applied. Moreover, arterial involvement is frequently overestimated on preoperative CT [17]. Given this diagnostic complexity, perineural invasion of the arterial perineural tissue has been established as a surrogate marker of perivascular arterial invasion comparable with arterial invasion, as demonstrated in a large multicenter study of 13 centers and over 1000 patients [15].

While the role of CT in PDAC staging is well established, there remains a need for continued optimization of its diagnostic performance in contemporary clinical practice. The current literature comparing CT with both intraoperative and histopathological findings remains scarce. This difficulty arises from the fact that the NCCN-criteria constitute a grading system for clinical decision making, deciding between three degrees, whereas surgery and histopathology use binary classification systems. However, understanding the degree of concordance between CT-based assessment and actual intraoperative findings with histopathological confirmation is essential for refining imaging protocols, improving radiological reporting standards, and optimizing patient selection for surgery. The temporal interval of preoperative imaging is also critical for the management of PDAC. Yet, German guidelines do not provide specific recommendations in this regard [18].

This retrospective study aimed to systematically compare preoperative contrast-enhanced CT findings with intraoperative and histopathological assessment of vascular involvement in patients with PDAC who underwent primary surgery.

## Materials and methods

### Study design and patient characteristics

This single-center, retrospective cohort study was conducted at a tertiary center with a HPB-specialized focus following approval from the ethics committee of the Medical Association Westfalia-Lippe (2025-506-f-S). The requirement for informed consent was waived because of the retrospective nature of the study.

**Fig. 1:**
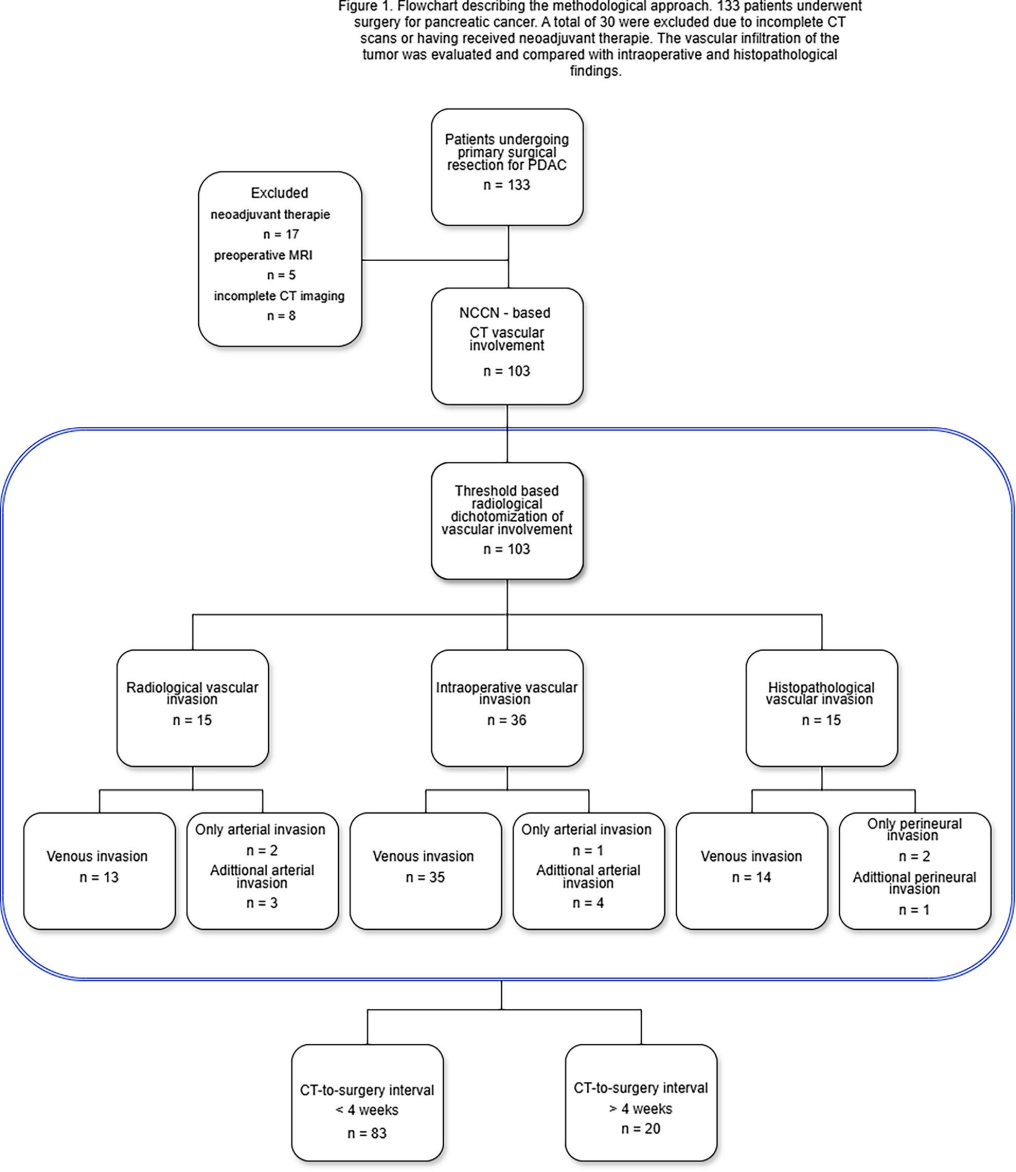
Flowchart describing the methodological approach. One hundred thirty-three patients underwent surgery for PDAC. A total of 30 were excluded due to incomplete CT scans or having received neoadjuvant therapy. The vascular infiltration of the tumor was evaluated and compared with intraoperative and histopathological findings

556 patients were presented in our hospital with a suspected diagnosis of PDAC. All patients underwent standardized contrast-enhanced CT-scan, the accepted reference standard for PDAC diagnosis, an endoscopic ultrasonography, and laboratory tests (clinical chemistry with bilirubin, lipase, gammaGT) with tumor marker (CA19-9). According to the international/national guideline, patients are classified as having resectable, borderline resectable, or unresectable disease based on vascular involvement, tumor biology, and Eastern Cooperative Oncology Group performance status. Primary standard surgery was offered to patients with resectable PDAC in accordance with the German S3-guidelines [18], as well as NCCN-criteria [4], within the framework of the interdisciplinary institutional tumor board (Fig. 1).

The inclusion criteria were as follows: (a) diagnosed PDAC; (b) complete preoperative contrast-enhanced CT performed with arterial and portal venous phase acquisitions; (c) primary surgical resection without neoadjuvant therapy; (d) detailed documentation of intraoperative and histopathological findings.

The exclusion criteria were as follows: (a) patients who received neoadjuvant chemotherapy (*n* = 17); (b) exclusively preoperative magnetic resonance imaging (MRI) (*n* = 5); (c) CT examinations without an arterial contrast phase (*n* = 8); (d) incomplete surgical documentation (*n* = 0); and (e) incomplete pathological documentation (*n* = 0).

### Patients with a changed operative plan scenario

During surgery, resectability was assessed by evaluating the presence of hepatic or peritoneal metastatic disease. In addition, technical unresectability was assessed by exposure of the NCCN-relevant vessels. Conversion from minimally invasive to open surgery, who required intraoperative conversion from robot-assisted pancreaticoduodenectomy to open procedure due to technical considerations or intraoperative findings, was documented.

### CT imaging protocol

All examinations used dedicated pancreatic protocol CT scans on Siemens multidetector CT scanners (Sensation 40, Somatom X.cite, Somatom Definition Flash). Non-ionic contrast (350–370 mg I/mL, 100–150 mL at 3–4 mL/s) was administered with bolus tracking for the arterial phase (threshold 100–150 HU in abdominal aorta, typically 25–35 s post-injection) and fixed delay for portal venous phase (60–70 s). While scanner technology evolved from 2012 to 2023, core protocol parameters remained consistent. Axial slice thickness was 1-3 mm, with 1 mm multiplanar reconstructions (MPR) routinely performed in coronal, sagittal, and axial planes for vascular assessment.

### NCCN-derived CT vascular invasion assessment

**Fig. 2:**
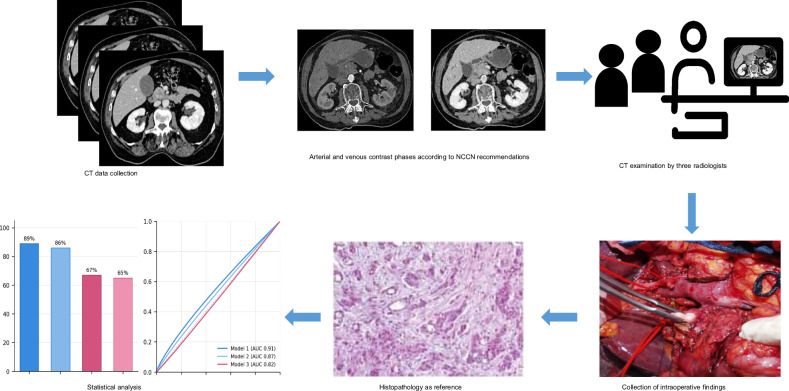
Workflow describing the methodological approach. CT scans with arterial and venous contrast phase were downloaded. Vessel invasion was examined by three radiologists. Intraoperative findings were collected, as well as histopathological data. Statistical analysis was performed

All CT examinations were independently reviewed by three radiologists with five (Z.M.), six (G.N.M.), and twelve years of experience in pancreatic imaging (G.H.P.) (Fig. 2). In cases of disagreement, a consensus was reached by majority vote. All readers were blinded to the intraoperative and histopathological findings. Vascular invasion was defined by a combination of established radiological criteria, encompassing morphological alterations of the PV and SMV. These included the “tear drop sign”, characterized by a distinctive narrowing and tapering deformity of the venous lumen, as well as contour irregularity reflecting direct tumoral abutment or encasement of the vessel wall, and intraluminal thrombosis as an indicator of advanced venous involvement. All features were systematically evaluated on contrast-enhanced CT in multiplanar, thin-sliced reconstructions. Venous vascular involvement was classified using the NCCN criteria [4] based on the degree of circumferential contact between the tumor and vessel: (a) no contact; (b) contact without vessel deformity; (c) contact with vessel narrowing or contour irregularity; and (d) vessel occlusion. Based on these criteria, each case was classified into: resectable, borderline resectable or locally advanced (unresectable).

### Threshold-based CT venous vascular invasion determination

For venous concordance analysis with intraoperative and histopathological findings, following previous studies [17], radiological vascular infiltration was dichotomized as “invasion” (> 180° or contour irregularity) vs “no invasion” (no contact or contact ≤180° without contour irregularity) between the tumor and each assessed vessel (Table 1). This binary classification was necessitated by the dichotomous nature of our reference standards: intraoperative findings and histopathological examination determine vascular invasion as either present or absent, enabling direct comparison between modalities.

**Table 1:** Dichotomization of venous infiltration in comparison to the NCCN-criteria for concordance analysis with histopathology (A); and dichotomization in comparison to the NCCN-Criteria with arterial Pn-Status as surrogate marker for histopathological estimation (B)

Vessel site	NCCN criteria	CT-based vessel invasion dichotomization	Histopathological vessel invasion assessment
1A. Venous assessment			
Venous	No contact to SMV or PV	No invasion	No invasion (V0)
	≤ 180° without contour irregularity		
	> 180° solid tumor contact to SMV or PV, ≤ 180° with contour irregularity or local thrombosis	Invasion	Invasion (V1: microscopic intraluminal tumor; V2: macroscopic)
	Solid tumor contact to inferior vena cava		
	Non-reconstructable SMV or PV in cases of tumor involvement or occlusion		
2B. Arterial assessment using Pn-Status surrogate marker for histopathological estimation			
Arterial	No contact to CA, SMA or CHA	No invasion	No invasion (Pn0)
	Solid tumor contact with CHA without extension to CA or CHA bifurcation	Invasion	Perineural arterial Invasion (Pn1)
	Solid tumor contact to SMA / CA ≤ 180° or solid tumor contact to anatomical variant		
	Solid tumor contact to CA > 180° wihtout involvement of the aorta and without involvement of gastroduodenal artery		
	Solid tumor contact to SMA / CA > 180° or solid tumor contact to CA with involvement of the aorta		

*NCCN* national comprehensive cancer network, *SMV* superior mesenteric vein, *PV* portal vein, *CA* celiac axis, *SMA* superior mesenteric artery, *CHA* common hepatic artery

### Intraoperative venous vascular invasion assessment

Intraoperatively, the presence or absence of vascular infiltration in each major peripancreatic vessel during surgical exploration was documented. Vascular involvement was defined as tumor adherence to or invasion of the vessel wall, requiring vascular resection/reconstruction, or precluding complete resection. Venous resection status influenced pathologic ascertainment: resected venous segments underwent direct histopathological examination of the vessel wall, while cases without venous resection were assessed based on adventitial tissue adherent to the tumor specimen.

### Histopathological venous vascular invasion assessment

All resected specimens underwent comprehensive histopathological examinations following standardized protocols. The microscopic and macroscopic presence or absence of vascular infiltration was documented. The resection margin status was classified as R0 (tumor-free resection margins with clearance ≥ 1 mm), R1 (microscopic residual tumor at the resection margin with clearance < 1 mm), or R2 (macroscopic residual tumor). Venous resection specimens were marked with sutures by the surgeon, underwent frozen section margin assessment, and were processed with standard paraffin embedding and two-level sectioning. Venous invasion (V1) was defined according to UICC TNM criteria [19], with definitive V1 classification requiring tumor cells in the vessel lumen; tumor cells in the vessel wall showed inter-pathologist interpretation variability. V1 status encompasses microscopic invasion of any venous vessel, including small vessels that cannot be detected on CT imaging. Therefore, to ensure comparability with radiological assessment, we classified patients as V1 only when microscopic invasion of major vessels (PV or SMV) was found and explicitly documented in the histopathological report.

### Threshold based CT arterial vascular invasion determination

Arterial involvement, as well as venous involvement, was radiographically classified using the NCCN-criteria based on the degree of circumferential contact between the tumor and artery: (a) no contact; (b) tumor abutment (contact ≤ 180° of the vessel circumference); or (c) tumor encasement (contact > 180° of the vessel circumference) and dichotomized (Table 2).

**Table 2:** Baseline demographic and clinical characteristics of study population

Parameter	Patients (*n*)	%	CT-Surgery Interval ≤ 4 weeks (*n* = 83)	CT-Surgery Interval > 4 weeks (*n* = 20)	*p*-value
Demographics					
Age (years)	103		68 (59–76)	65 (53–74)	0.10^a^
Gender (male)	63	61	48 (58%)	15 (75%)	0.52^b^
Body mass index (kg/m²)	102		24 (22–26)	24 (22–29)	0.15^b^
Tumor location					
Pancreatic head/uncinate process	76	74	64 (77%)	12 (60%)	0.76^b^
Pancreatic body/tail	19	18	15 (18%)	4 (20%)	
Multiple locations	8	8	4 (5%)	4 (20%)	
NCCN resectability classification (CT-based)					
Resectable	86	84	73 (88%)	13 (65%)	0.18^b^
Borderline resectable	17	16	10 (12%)	7 (35%)	
Unresectable	0	0	0	0	
Surgical procedure					
Pancreatic head resection	57	55	51 (61%)	6 (30%)	0.01^b^
Pancreatectomy	30	29	19 (23%)	11 (55%)	
Distal pancreatic resection	16	16	13 (16%)	3 (15%)	
Histopathological status					
R0 (margin negative)	74	69	63 (75%)	11 (55%)	0.20^b^
R1 (margin positive)	29	28	20 (25%)	9 (45%)	
V0 (no venous vessel invasion)	89	86	73 (88%)	16 (80%)	0.76^b^
V1 (microscopic venous vessel invasion)	13	13	10 (12%)	3 (15%)	
V2 (macroscopic venous vessel invasion)	1	1	0 (0%)	1 (5%)	
Pn0 – microscopic surrogate marker for arterial vessel invasion	74	72	16 (19%)	2 (10%)	0.68^b^
Pn1– microscopic surrogate marker for arterial vessel invasion	29	28	67 (81%)	18 (90%)	

Continuous variables are presented as medians (interquartile range). Categorical variables are presented as frequencies (percentages)

*NCCN* national comprehensive cancer network, *CT* computed tomography

^a^ *T*-test for independent samples

^b^ *p*-values derive from McNemar test for paired categorical data

### Intraoperative arterial vascular invasion assessment

Analogously to venous vessels, arterial invasion was intraoperatively defined as tumor adherence to or invasion of the vessel wall and accordingly documented.

### Histopathological arterial vascular invasion estimation

According to UICC TNM criteria [19], arterial invasion requires tumor cells in the arterial lumen, with wall involvement showing inter-pathologist variability; perineural or soft tissue infiltration adjacent to arteries does not constitute arterial invasion. Major arteries remain in situ during standard pancreaticoduodenectomy and are not available for histopathological examination. Therefore, histopathological assessment was limited to periarterial soft tissue examination and could not confirm or exclude true arterial wall invasion. Periarterial Perineural invasion (Pn_art_) status was systematically assessed in all specimens according to UICC TNM criteria (Pn0: absent; Pn1: present). Although perineural invasion adjacent to arteries does not constitute arterial lumen invasion, it may serve as a surrogate marker for advanced local tumor extension near vascular structures.

### Statistical analysis

Statistical analyses were performed using the IBM SPSS Statistics software (Version 31.0). Continuous variables were expressed as medians with interquartile ranges (IQRs) (Q1–Q3) given their non-normal distribution, while categorical variables were presented as frequencies and percentages. Inter-reader agreement for CT-based vascular invasion assessment was evaluated among all three readers using Fleiss’ kappa (κ). The concordance between radiological-intraoperative assessment of vascular involvement was evaluated using McNemar’s test for paired categorical data. The concordance between radiological-histopathological assessment of vascular involvement was evaluated using McNemar’s test for paired categorical data. Sensitivity was defined as the proportion of surgically confirmed vascular invasions correctly identified on CT (true-positive rate). Specificity was defined as the proportion of vessels without surgical invasion that were correctly classified as uninvolved on CT (true-negative rate). A subgroup analysis was performed to evaluate the impact of the temporal interval between the CT and surgery on diagnostic performance using Cohen´s Kappa and McNemar’s test for paired categorical data. The 4-week (28-day) threshold for CT-to-surgery interval was selected a priori based on previous studies reporting optimal intervals of 25 days [20, 21]. This threshold represents approximately one-fifth of the mean tumor volume doubling time reported by Ahn et al [22], a timeframe sufficient for detectable progression in rapidly proliferating tumors. A two-sided *p*-value < 0.05 was considered statistically significant for all comparisons.

## Results

Patient characteristics A total of 103 patients (63 men, 61%) with a median age of 68 years (IQR: 59–75 years) underwent primary surgical resection for PDAC during the study period. The majority of tumors were located in the pancreatic head or uncinate process (*n* = 76, 74%). The median body mass index was 24 kg/m² (IQR: 22–26.3 kg/m²). Based on preoperative CT assessment according to the NCCN-criteria, 86 patients (84%) were classified as resectable, while 17 patients (16%) were categorized as borderline resectable. No patients were classified as unresectable. Patient characteristics are demonstrated in Table 2. In the subgroup with a shorter CT-to-surgery interval ( ≤ 4 weeks), pancreatic head resection was the most frequently performed procedure, whereas total pancreatectomy was less common (*p* = 0.01). All other demographic characteristics were comparable between patients undergoing surgery ≤ 4 weeks and > 4 weeks after CT (all *p* > 0.1)

### Patients with changed operative plan scenario

Conversion from minimally invasive to open surgery was found in 2 patients (2%) who required intraoperative conversion from robot-assisted pancreaticoduodenectomy to open procedure due to technical considerations or intraoperative findings. 29 patients (28%) demonstrated more extensive local disease than anticipated on preoperative imaging, including unexpected involvement of adjacent organs, more extensive lymph node involvement, or greater tumor size than estimated radiologically, resulting in unexpected R1 resection status in some cases. 28 patients (27%) underwent PV and/or SMV resection with reconstruction when intraoperative assessment confirmed venous involvement that was technically amenable to reconstruction.

### NCCN-derived CT vascular invasion interobserver agreement

**Fig. 3:**
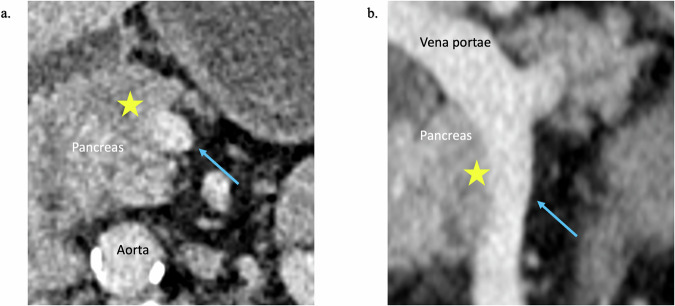
CT scan (**a** venous contrast phase axial, **b** venous contrast phase coronal) of an 82 years old male patient with pancreatic adenocarcinoma of head, pT2 pN2 (9/34, ECE+) L1 V1 Pn1 R0. Discrepant radiological opinion regarding venous vessel invasion (star = tumor, blue arrow = SMV)

Concordance analysis across all observers revealed high agreement for venous vessel invasion (κ = 0.8) and substantial agreement for arterial vessel invasion (κ = 0.6) (Fig. 3).

### Threshold based CT venous vascular invasion determination

On preoperative CT, 15 patients (15%) showed vascular involvement, of whom 13 patients (13%) showed venous infiltration (Table 3).

**Table 3:** CT-based venous invasion assessment using NCCN-criteria and operational dichotomized definition of vascular invasion and concordance analysis between different modalities

Prevalence	*n*	%
CT	13	13
Intraoperative findings	35	34
Histopathological findings	15	15

Intermodality correlation	Concordance rate (%)	Sensitivity/specificity (%)
CT vs surgery	75	31/97
CT vs histopathology	81	27/90

The rates represent cases with observed venous vessel invasion in the assessment modalities. Concordance rates represent the proportion of cases with agreement between the assessment modalities and was derived from the McNemar test for paired categorical data

*CT* computed tomography

### Intraoperative venous vascular invasion assessment

Intraoperatively, 36 patients (35%) showed vascular involvement, 35 of whom (34%) with venous involvement (Table 3). Venous resection with reconstruction was performed in 34% of patients, when venous involvement was confirmed intraoperatively and reconstruction was technically feasible.

### Histopathological venous vascular invasion assessment

**Fig. 4:**
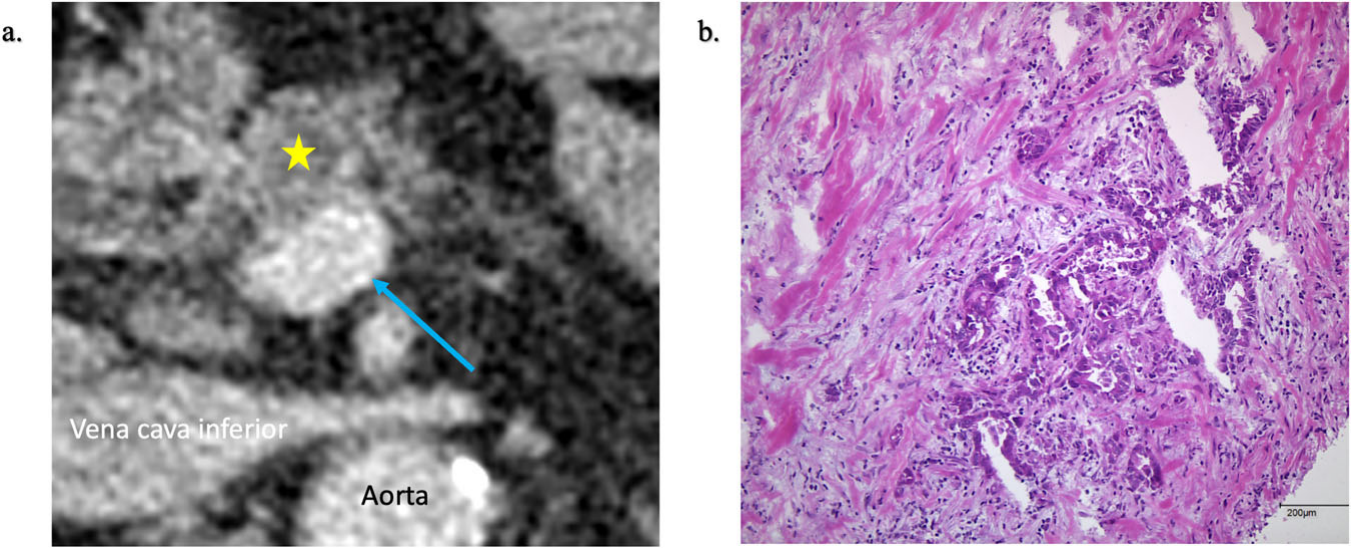
**a** CT scan with venous contrast phase of a 85 years old male patient with pancreatic adenocarcinoma of head and body. Radiological infiltration of the SMA (star = tumor, blue arrow = SMV). **b** Histopathological specimen showing infiltration of the SMV. Irregular glands in the vessel wall

Histopathological examination revealed R0-Status in 74 (69%) and R1-Status in 29 patients (28%). The V-Status was V0 in 89 patients (86%), V1 in 13 (13%) and V2 in 1 patient (1%) (Table 3 and Fig. 4).

### CT-surgery venous invasion concordance

**Fig. 5:**
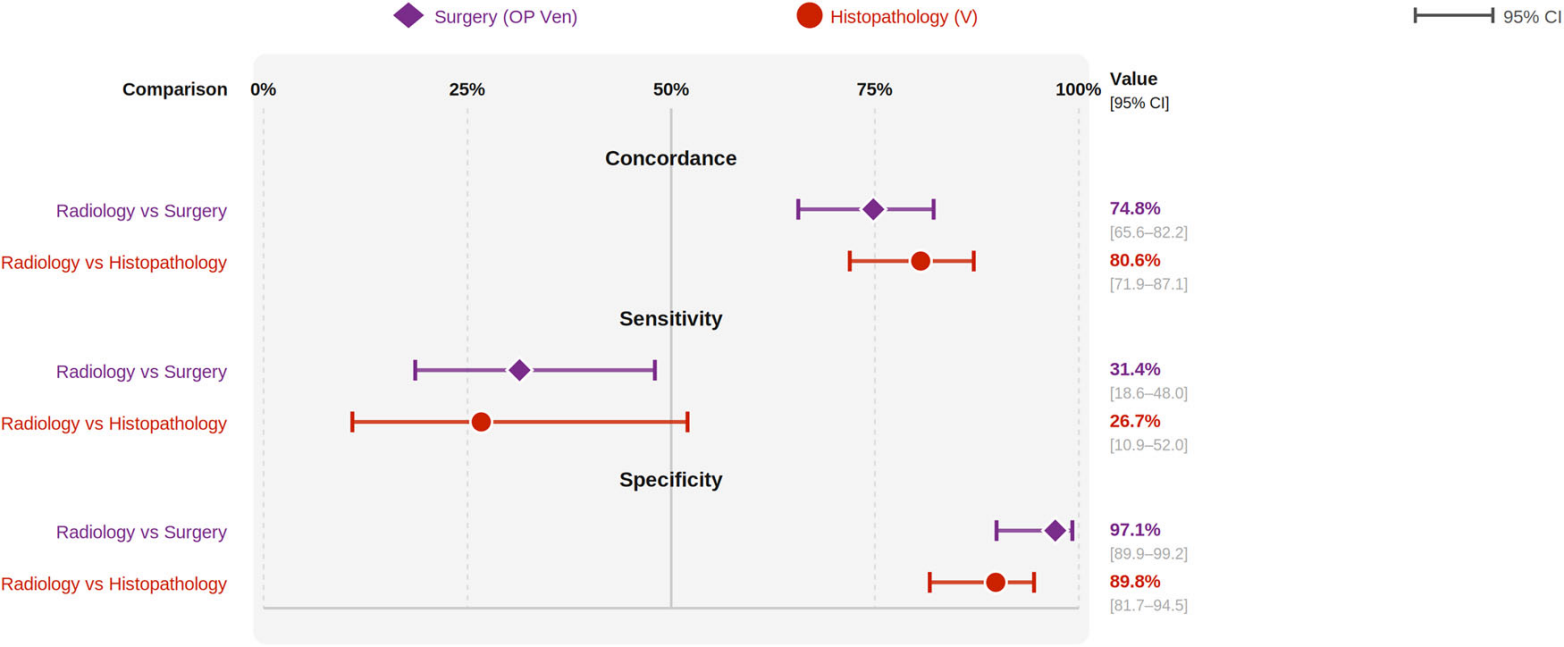
Concordance analysis of venous invasion between different assessment modalities. Forest plot showing sensitivity and specificity with 95% confidence intervals for venous vascular territories. *N* = 103 patients

Compared with surgical findings, CT showed a sensitivity of 31% (95% confidence interval (CI): 19–48) and specificity of 97% (95% CI: 90–99), with a concordance rate of 75%, indicating statistically significant difference (*p* < 0.001) (Table 3 and Fig. 5).

### CT-Histopathology venous invasion concordance

The concordance between radiological CT and histopathological findings in venous vessels was 81% (sensitivity, 27%, 95% CI: 11–52; specificity, 90%, 95% CI: 82–95), demonstrating no significant difference (*p* = 0.82) (Table 3 and Fig. 5).

### Arterial and arterial perineural (perivascular) invasion estimation

**Fig. 6:**
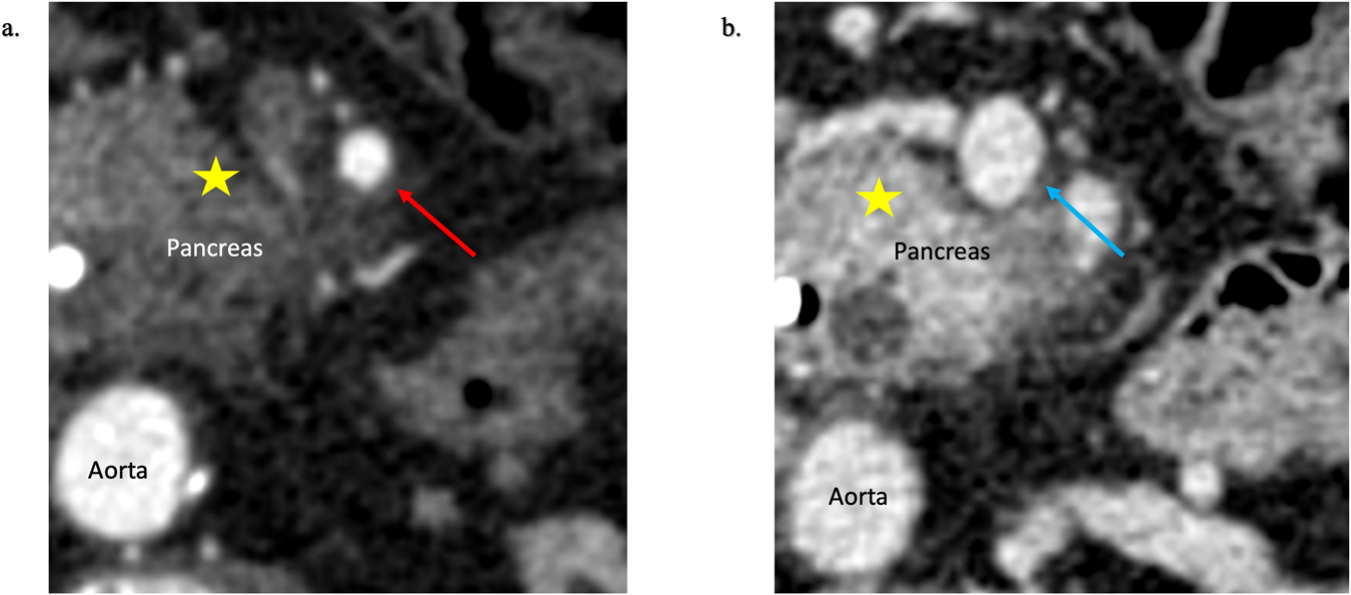
CT scan (**a** arterial contrast phase, **b** venous contrast phase) of an 80 years old female patient with pancreatic adenocarcinoma of head, pT2 pN0 (0/23) L0 V0 Pn1 R1 M0. Radiological infiltration of the SMA, with no macroscopic invasion intraoperatively and arterial Pn0 status of surrogate marker histopathologically, however as a limitation, due to SMA not being resected (star = tumor, red arrow = SMA, blue arrow = SMV)

5 patients (5%) demonstrated arterial infiltration on CT. Intraoperatively, 5 patients (5%) showed arterial involvement, of whom 1 had only arterial invasion and 4 additional arterial and venous invasion. Compared with the intraoperative assessment of arterial vessels, CT demonstrated a sensitivity of 60% (95% CI: 23–88) and specificity of 98% (95% CI: 92–99), resulting in an overall concordance of 96% (*p* = 1.0), indicating high agreement with no statistically significant difference (Fig. 6). Histopathologically no patient had microscopic arterial invasion. Periarterial perineural invasion (Pn_art_1) was identified in the periarterial tissue of major arteries (SMA/CHA) in 3 patients (3%), of whom 2 had only perineural invasion and 1 additional perineural and venous invasion. While this does not represent arterial lumen invasion, it indicates tumor extension into the arterial neural plexus. The concordance between radiological CT and histopathological findings in arterial vessels was 95% (sensitivity 0%, 95% CI: 0–79; specificity 95%, 95% CI: 89–98), demonstrating no significant difference (*p* = 0.82) (Table 4 and Fig. 5). When analyzing periarterial perineural invasion, CT showed 96% concordance with arterial Pn_art_1-status (sensitivity 67%, 95% CI: 9–99, specificity 97%, 95% CI: 92–99%, *p* = 0.62). All sensitivity and specificity values reflect the dichotomized definition of vascular invasion compared to histopathological estimation.

**Table 4:** CT-based arterial invasion assessment using NCCN-criteria and operational dichotomized definition of vascular invasion compared with surgical findings and histopathological estimation with Pn-Status as surrogate marker

Prevalence	*n*	%
CT	5	5
Intraoperative findings	5	5
Histopathological findings	3	3

Intermodality correlation	Concordance rate to surgery (%)	Sensitivity/specificity of results to the dichotomized definition of vascular invasion compared to surgery and to histopathological estimation (%)
CT vs surgery	96	60/98
CT vs histopathology	96	67/97

The rates represent cases with observed arterial vessel invasion on CT and surgery, as well as the perineural arterial invasion histopathologically. Concordance rates represent the proportion of cases with agreement between the assessment modalities and was derived from the McNemar test for paired categorical data

*CT* computed tomography

### Comparison of CT-imaging interval to surgery and histopathology

In the ≤ 4 weeks group, venous assessment achieved a concordance with surgical findings of 77%, with a sensitivity of 27% (95% CI: 14–46%) and specificity of 100% (95% CI: 94–100%) (*p* = 0.5). In patients with longer intervals (> 4 weeks), venous concordance decreased to 65%, with a sensitivity of 50% (95% CI: 24–76%) and specificity of 80% (95% CI: 49–94%) (*p* = 0.4). Similarly, in the ≤ 4 weeks group, venous assessment achieved concordance with histopathological findings of 84%, with a sensitivity of 20% (95% CI: 6–51%) and specificity of 93% (95% CI: 85–97%) (  = 0.5). In patients with longer intervals (> 4 weeks), venous concordance decreased to 60%, with a sensitivity of 40% (95% CI: 12–77%) and specificity of 67% (95% CI: 42–85%) (*p* = 0.7) (Table 5).

**Table 5:** Diagnostic performance of CT for venous invasion stratified by CT-to-surgery interval

CT-to-surgery interval	CT imaging ≤ 4 weeks (*n* = 83)	CT imaging > 4 weeks (*n* = 20)
CT vs intraoperative venous assessment		
Concordance rate to the definition of vascular invasion	77%	65%
Sensitivity	24% (14–46%)	50% (24–76%)
Specificity	100% (94–100%)	80% (49–94%)
* p*-value	0.5	0.4
CT vs histopathological venous assessment		
Concordance rate to the definition of vascular invasion	84%	60%
Sensitivity	20% (6–51%)	40% (12–77%)
Specificity	93% (85–97%)	67% (42–85%)
* p*-value	0.5	0.7

Concordance rates represent the proportion of cases with agreement between radiological, surgical and histopathological findings. Concordance rate and *p*-values derived from McNemar test for paired categorical data

*CT* computed tomography

## Discussion

In this interdisciplinary study, we evaluated interobserver agreement of NCCN-derived CT venous involvement compared to surgery and histopathology in 103 therapy-naïve PDAC patients undergoing primary surgery. Additionally, we evaluated CT arterial status to surgery with periarterial perineural invasion (Pn_art_) as histopathological estimation in a few patients. To enable interdisciplinary agreement analysis, graded NCCN-derived radiological vascular infiltration parameters were operationally dichotomized and defined as invasion (> 180° circumferential contact or contour irregularity) vs no invasion (no contact or ≤ 180° contact without contour irregularity). The project was necessitated by the scarce interdisciplinary studies, due to the complexity of PDAC staging.

Interobserver radiological agreement for both venous and arterial CT-based vascular invasion assessment was strong in the present study, consistent with previously reported studies [12–14]. The comparatively lower agreement observed in some other studies may be attributable to the inclusion of patients with neoadjuvant therapy, in whom treatment-induced stromal fibrosis, edema, and inflammation are known to substantially confound radiological vascular assessment and lead to systematic overestimation of vascular invasion [23].

In our study the overall incidence of 24% of PDAC patients undergoing upfront surgery at the time of diagnosis is similar to previous studies [24, 25]. The predominance of venous invasion observed in the present cohort is consistent with the established histopathological literature, in which venous invasion has been reported in up to 65–88% of surgically resected PDAC [26, 27].

CT-surgery venous invasion concordance demonstrated high specificity (97%) but low sensitivity (31%), consistent with previously reported sensitivity ranges of 14–54% and specificity of 75–95% [28], confirming the tendency of CT to overestimate true venous involvement while reliably excluding invasion when absent. CT-histopathology venous invasion concordance demonstrated high specificity (90%) but low sensitivity (27%), consistent with the pattern reported in the literature, in which CT specificity against histopathology is consistently high (94–98%) while sensitivity remains lower [15, 29].

CT-to surgery arterial invasion concordance showed high specificity (98%) with moderate sensitivity (60%), consistent with established patterns of CT reliability excluding arterial involvement when absent while incompletely capturing true arterial invasion [16].

The CT-histopathology concordance analysis for arterial involvement in the present study was necessarily restricted to periarterial perineural invasion, as none of the included patients met criteria for Appleby-type arterial resection. Noda et al [16] performed a direct CT-to-histopathology comparison of arterial invasion; however, their analysis focused on the splenic artery, which falls outside the scope of NCCN-resectability criteria and is therefore not directly applicable to the clinical context of the present study. Although Egorov et al [30] reported an absence of true arterial wall involvement in patients with periarterial perineural invasion, the use of perineural invasion status as a surrogate marker for arterial involvement in the present study is rationalized by, and consistent with, a large multicenter study comprising over 1000 patients across more than 30 centers, which applied the same surrogate approach [15]. To the best of our knowledge, this interdisciplinary study is the first to systematically investigate each intermodal definition of vascular invasion focusing on detailed histopathology, as applied to different classification systems across radiology, surgery, and histopathology, thereby addressing a critical methodological gap in the existing literature.

The time interval between imaging and surgery is crucial for precise preoperative radiological evaluation, as we identified a 24% deterioration in concordance rate for venous involvement in the CT-to-surgery interval exceeding 4 weeks. This temporal dependency has received limited attention in the literature but has significant clinical implications. Ahn et al [22] demonstrated that PDAC exhibit highly variable growth rates, with volume doubling times ranging from 20 to 976 days, suggesting that substantial tumor progression can occur within a 4-week interval in rapidly growing tumors. Our findings complement a recent study by Healy et al [20], who reported that performing surgery within 25 days of CT reduced the risk of unexpected disease progression.

The integration of advanced imaging techniques such as photon-counting CT may further improve the reliability of vascular invasion assessment in PDAC. Photon-counting CT has demonstrated substantially better inter-reader agreement for peripancreatic vessel involvement compared to conventional energy-integrating detector CT, alongside a significant reduction in radiation dose [31]. Further prospective studies should evaluate whether this advantages translate into more consistent resectability classification, particularly in borderline resectable cases. Quantitative MRI [32] and radiomics-based quantitative analysis [33], may also improve the detection of vascular invasion and reduce the interpretation variability.

This study has several limitations. Our cohort consisted exclusively of therapy-naive patients who underwent standard surgical resection, introducing selection bias. While this decision was methodologically necessary, as cases without surgery lack complete intraoperative vascular assessment and histopathological specimens required for concordance analysis, it may result in upward estimation of CT performance. Additionally, the present study focused on patients undergoing upfront surgery, and the applicability of CT-based vascular assessment criteria in the post-neoadjuvant setting remains a distinct challenge [34]. While refined CT criteria incorporating contact length and contour deformity have shown promising diagnostic performance in borderline resectable patients after neoadjuvant therapy [35], the reliability of the > 180° encasement threshold in this context remains limited [36]. Whether the vascular assessment criteria evaluated in our study maintain their diagnostic performance in post-neoadjuvant cohorts warrants dedicated investigation, which our group intends to address in a subsequent study.

A further limitation relates to the sensitivity of the results to the operational definition of vascular invasion. The decision to classify the radiological vessel involvement in any degree of contact as invasion to applying NCCN-aligned thresholds altered the observed diagnostic performance, indicating that the findings are, at least in part, threshold-dependent rather than solely reflective of intrinsic CT diagnostic capability. This limitation is inherent to the application of a binary classification framework to an essentially graded radiological variable: whereas CT-based vascular assessment is continuous in nature and clinical decision-making follows NCCN-categories, histopathological and intraoperative reference standards are fundamentally binary. The present study therefore quantifies agreement between a specific dichotomization strategy and binary reference standards, and the generalizability of the findings is contingent on the chosen definitional framework. Further, the observed reduction in venous concordance in the subgroup analysis may be attributable to the small number of patients with CT-to-surgery intervals exceeding 4 weeks. Finally, the diagnostic performance metrics reported for arterial involvement do not represent true accuracy for arterial wall invasion in the conventional sense, as direct histopathological confirmation of major peripancreatic arterial infiltration is not feasible in standard resection specimens. Arterial performance metrics were therefore derived using periarterial perineural invasion (Pn_art_1) as a surrogate histopathological reference standard, an approach supported by prior methodology but inherently limited by the imperfect correlation between perineural and direct mural infiltration. Accordingly, arterial concordance values should be interpreted with caution and may not be directly comparable to venous performance metrics, for which true histopathological confirmation was available.

In conclusion, NCCN-derived, threshold based radiological venous invasion determination is similar to binary intraoperative and histopathological findings and decreased with longer CT-to-surgery intervals, showing the need for establishing timeframes for preoperative imaging.

## Data Availability

The raw data supporting the findings of this study are available from the corresponding author upon reasonable request. Due to patient privacy protection regulations and institutional data protection policies in accordance with GDPR, individual patient data cannot be made publicly available. Researchers requesting access to the data must provide a methodologically sound proposal, obtain approval from their local ethics committee, and sign a data sharing agreement with the University Hospital Münster. Aggregated data presented in the tables and figures are available within the manuscript.

## Abbreviations

CA: Celiac axis
CHA: Common hepatic artery
CI: Confidence Interval
IQR: Interquartile range
PDAC: Pancreatic ductal adenocarcinoma
PV: Portal vein
SMA: Superior mesenteric artery
SMV: Superior mesenteric vein
